# The relationship between job components, neighbourhood walkability and African academics' physical activity: a post-COVID-19 context

**DOI:** 10.1093/heapro/daab090

**Published:** 2021-07-19

**Authors:** Nestor Asiamah, Frank Frimpong Opuni, Faith Muhonja, Emelia Danquah, Simon Mawulorm Agyemang, Irene Agyemang, Akinlolu Omisore, Henry Kofi Mensah, Sylvester Hatsu, Rita Sarkodie Baffoe, Eric Eku, Christiana Afriyie Manu

**Affiliations:** 1 University of Portsmouth, School of Health and Care Professions, Winston Churchill Ave, Portsmouth PO1 2UP, UK; 2 Africa Centre for Epidemiology, Accra Ghana, P. O. Box AN 16284, Accra North, Ghana; 3 Department of Marketing, Accra Technical University, Accra, Ghana; 4 School of Public Health, Mount Kenya University, Kenya; 5 Logistics and Supply Chain Management, Koforidua Technical University, Koforidua, Ghana; 6 Department of Physical Education and Health, Abetifi Presbyterian College of Education, Ghana; 7 Student Affairs, West African Postgraduate College of Pharmacists, Accra, Ghana; 8 Department of Community Medicine, Osun State University, Nigeria; 9 Human Resources and Organizational Development, Kwame Nkrumah University of Science and Technology, Kumasi, Ghana; 10 Department of Computer Science, Accra Technical University, Accra, Ghana; 11 School of Business, Accra Technical University, Accra, Ghana; 12 Department of Behavioural Epidemiology, University of Education, Winneba, Institute for Distance Education and E-learning, Winneba, Ghana

**Keywords:** teaching, research, assessment, physical activity, neighbourhood walkability

## Abstract

Research to date suggests that physical activity (PA) among academics is insufficient globally. Academics in many African countries were recently required to resume work while observing social distancing protocols. Physical inactivity (PI) was, therefore, expected to increase in such academics. Interestingly, walkable neighbourhoods are resources that could discourage excessive sitting and PI in this situation. This study, therefore, assessed the moderating role of neighbourhood walkability in the relationship between core job components (i.e. on-site teaching, online teaching, research and student assessment) and PA among academics. The study adopted a cross-sectional design that utilized an online survey hosted by Google Forms to gather data. Participants were volunteer full-time academics in Nigeria, Ghana, Kenya and Tanzania. A total of 1064 surveys were analysed, with a sensitivity analysis utilized to select covariates for the ultimate hierarchical linear regression model. After controlling for the ultimate covariates (e.g. gender, education and income), PA was found to be positively associated with the job component 'research work’ but negatively associated with student assessment. Neighbourhood walkability increased the positive relationship of research work with PA and reduced the negative relationship of student assessment with PA. The non-significant negative relationship between ‘teaching online’ and PA was made positively significant by neighbourhood walkability. We conclude that research as a job component is positively associated with PA, but online teaching is negatively associated with PA among African academics in a post-COVID-19 context.

## INTRODUCTION

A plethora of studies have reported a significant decline in physical activity (PA) and exercise as a result of Coronavirus 2019 (COVID-19) social distancing measures (Armitage and Nellums, 2020; Asiamah *et al.*, 2021; Violant-Holz *et al.*, 2020). This situation is the result of a loss of PA time due to limited access to social ties and neighbourhood services in the general population (Asiamah *et al.*, 2021). At the national and global levels, the closure of universities is outstanding amongst COVID-19 containment strategies (Adarkwah, 2020; Sahu, 2020). For two main reasons, university faculties may have faced an unprecedented level of public health risk due to this development. First, many academics in Africa and other regions had to live in isolation with limited access to neighbourhood and social resources (Mahmood, 2020; Rajhans *et al.*, 2020; Sahu, 2020; Upoalkpajor and Upoalkpajor, 2020). Before this development, teaching in a physical classroom was the only PA-oriented job task among academics (George *et al.*, 2014; Keenan and Greer, 2015), with the other two components (i.e. student assessment and research) being largely sedentary (Keenan and Greer, 2015). To illustrate, teaching onsite requires movement to a physical classroom through active forms of transportation such as walking and bicycling. Even if the individual drives to campus to teach, he or she would have to walk into the classroom and possibly walk around while teaching. While on campus to teach, academics may socialize with peers and students through social and physical activities. The other job components (i.e. online teaching, student assessment and research) require more hours of sitting. Online teaching, for example, involves sitting to instruct and observe students online, with little or no opportunity to walk. Marking and collation of examination scores, the main form of student assessment in Africa (Owolabi, 2020), require prolonged sitting. Research has two main components among academics, namely desk research (i.e. literature reviews) and field work (i.e. data collection) (Owolabi, 2020; Aczel *et al.*, 2021). Literature reviews are sedentary tasks because they require prolonged hours of sitting and reading. Data gathering in the field with questionnaires is an opportunity for walking and performing other forms of PA, but academics either outsource their data collection or task students to gather their data (Jacob, 2020; Owolabi, 2020). Thus, academics seldom engaged in field work. No doubt, sedentary time and physical inactivity (PI) increased because many academics took to online teaching and student assessment (Asiamah *et al.*, 2021; Violant-Holz *et al.*, 2020).

Research has shown that the use of computers for teaching is a leading cause of PI (Dhawan, 2020; George *et al.*, 2014; Keenan and Greer, 2015), which connotes that online teaching, sitting with screens (e.g. TV, phones and tablets) and related sedentary behaviours made academics less active as they observed social distancing protocols. Empirical studies have also indicated that academics generally spend up to 75% of their waking day in sedentary behaviour (George *et al.*, 2014; Keenan and Greer, 2015). The average daily sedentary behaviour time reported for academics before the outbreak of COVID-19 is about 8 h (Keenan and Greer, 2015), which is higher than the averages reported for the general population (Fountaine *et al.*, 2014). A report on global PA insufficiency levels (Guthold *et al.*, 2018) suggests that PA is at the highest level in Sub-Saharan Africa, which means that academics in this subregion may face a lower risk of PI. The same report revealed, however, that PA insufficiency is a global problem that needs to be addressed. It could be argued, therefore, that academics around the world were already inactive before the outbreak of COVID-19. As such, a higher risk of PI among academics attributable to COVID-19 responses is evident. Experts have opined that COVID-19 may take several months or years to contain and that the future cannot be free of similar pandemics (Armitage and Nellums, 2020; Asiamah *et al.*, 2021). COVID-19 has heralded an era when organizations and individuals may adopt social distancing strategies as a facet of work culture to save operational costs or reduce public health risks. As such, the above-mentioned changes in work patterns may increase the risk of PI among academics for the long term. Special PA interventions for university faculties and similar groups may, therefore, be urgently needed.

There is a consensus among researchers (Chudyk *et al.*, 2017; Christman *et al.*, 2019) that a major determinant of PA is neighbourhood walkability, defined as the extent to which built environment factors (e.g. services, crosswalks, sanitation, peace, etc.) support PA in the neighbourhood (Koohsari *et al.*, 2018). At any given time, neighbourhood walkability is a resource for keeping active (Dietz *et al.*, 2020; Pinheiro and Luís, 2020), which means that the neighbourhoods of academics can attenuate PI in an emerging culture of social distancing. It is, however, not clear if walkable neighbourhoods will be sufficiently utilized by academics to reduce PI when social distancing measures are still being enforced nationally or being adhered to at the individual level. Researchers (Jacob, 2020; Murphy, 2020) have reported that individuals continued to adhere to social distancing measures even in countries where COVID-19 restrictions were lifted or eased. By the close of August 2020, many African countries including Ghana, Nigeria, Tanzania and Kenya had their COVID-19 lockdowns lifted and universities reopened (Jacob, 2020; Upoalkpajor and Upoalkpajor, 2020). Some universities, in response, reintroduced physical classroom activities (e.g. on-site teaching) amidst more stringent social distancing measures (Jacob, 2020; Murphy, 2020). The reopening of universities also intensified online teaching, student assessment and research (Upoalkpajor and Upoalkpajor, 2020), which suggests that university faculties in these African countries still faced occupational barriers to PA. Thus, the reopening of universities in Africa required academics to resume work while observing more stringent national and university-level social distancing protocols. We refer to the time just after the reopening of universities as the post-COVID-19 era, a period when academics were required to perform core job tasks (i.e. teaching, student assessment and research) while observing social distancing protocols. With this development, an understanding of how the said job components are associated with PA among academics after the reopening of universities and easing of COVID-19 restrictions can benefit the development of special PA interventions for academics.

By easing or lifting COVID-19 restrictions, governments gave citizens more access to walkable neighbourhood factors (e.g. supermarkets, gyms, parks and transport stations) but that did not guarantee a significant use of neighbourhood services because social distancing protocols were still being implemented by service providers and individuals (Jacob, 2020; Upoalkpajor and Upoalkpajor, 2020). It is, thus, not clear if neighbourhood walkability can be savoured by academics to buffer PI after COVID-19 restrictions were eased. This study, therefore, assessed the relationship between academics' core job components (i.e. on-site teaching, online teaching, research and student assessment) and PA as well as the moderating role of neighbourhood walkability in this relationship. We focused on core job components of academics in Africa that are measurable during the pandemic and characterized most job tasks of academics. By assessing the relative associations of these job components with PA, universities and employers would understand how key academic job tasks correlated with PA during the pandemic. This understanding may prompt more resilient research designs (e.g. experimental designs) as well as policy and infrastructural interventions encouraging PA among academics.

What informed an assessment of this moderating role is the assumption that healthy behaviours (e.g. PA and social activity) are more modifiable in the short term if the immediate environments of individuals provide access to services and other walkable factors. Our moderating analysis can provide lessons for improving neighbourhood design and the provision of services to encourage PA in an atmosphere of COVID-19. Interventions encouraging PA in such an atmosphere are necessary as PA could be a way to boost immunity against infectious diseases (Armitage and Nellums, 2020; Asiamah *et al.*, 2021). Furthermore, neighbourhood walkability in Africa is generally low (Oyeyemi *et al.*, 2019; Asiamah *et al.*, 2020; Asiamah *et al.*, 2021), so moderating analyses such as the current one are needed to encourage policy action aimed at improving neighbourhood walkability. Finally, this study addresses a dearth of studies focused on PA in African academics (George *et al.*, 2014; Keenan and Greer, 2015) by answering two main research questions: (i) which of academics' core job components are associated with PA and (ii) does neighbourhood walkability moderate the possible relationship between the job components and PA? We attempted to answer these questions with data from four African countries associated with the said post-COVID-19 context.

## METHODS

### Design and approach

This study adopted a cross-sectional design and an online survey targeting academic staff of universities, polytechnics and colleges of education. The use of online surveys was the only way to collect data since the study was conducted at a time COVID-19 social distancing measures were still being observed.

### Study setting, population and selection

The study setting was African countries (i.e. Ghana, Nigeria, Tanzania and Kenya) where tertiary institutions reopened by 31 August 2020, and followed more stringent national social distancing protocols in their operational activities. The study population was the academic staff of universities in these countries. Participants were selected based on six criteria: (i) being a full-time academic; (ii) having at least a year of work experience as an academic; (iii) having a minimum of a diploma, which was an indicator of the ability to read and write in English, the medium in which surveys were administered; (iv) resumption of one or more job duties after the reopening of universities; (v) observance of national or institutional social distancing protocols; and (vi) willingness to participate in the study. We could not use a powered sample (i.e. a sample determined based on pre-calculated effect size and power) because no related study with relevant statistics (e.g. effect size) was available. Recent COVID-19 studies and previous online surveys (Asiamah *et al.*, 2021; Elsahory *et al.*, 2020) had used sample sizes ranging between 32 and 4222 to reach useful findings. Considering our study design and the geographical scope of our study, we aimed to collect data from a sample of 1000 academics who met the above selection criteria.

### Structure of the survey

The online survey was developed by the researchers and hosted on Google Forms, a free survey creation platform that allows data sharing between research collaborators. We chose Google Forms over other related tools because it is user-friendly, affordable and allows easy transfer of data to the chosen statistical software. Because no existing template was suited to our study, we developed the survey from scratch. The survey comprised five sections, with the first section made up of the first two questions. The first question presented the instructions for completing the survey and ethics statement (i.e. participant consent statement). The second question screened for only volunteer participants. With this question, we ensured that only individuals who agreed to participate in the study by ticking ‘Yes’ could complete the survey. The second section presented seven items measuring the core job components, namely on-site teaching, online teaching, research and student assessment. Sections 3 and 4 captured measures of neighbourhood walkability and PA, respectively. Section 5 presented 12 demographic and individual characteristics. We applied the ‘multiple questions per page’ online survey format because the other format (i.e. one question per page) would have made our survey too long. The literature indicates that the use of excessively long surveys results in a low response rate (Sahlqvist *et al.*, 2011).

### Development and validation of the survey

To develop and validate the survey, we employed the robust procedure of Asiamah *et al.* (Asiamah *et al.*, 2021). In this vein, the researchers discussed what could be the most suitable measures of PA, neighbourhood walkability and the job components with three groups. The first group, which included four of the researchers, was a WhatsApp-based group of Research Associates at a Center of Excellence. All members of this group were academics with ranks ranging from Lecturer to Associate Professor. Members suggested scales or measures perceived to fit the study context. Over Zoom, the researchers consulted with the second group (comprising three statisticians and two psychometricians from Ghana, USA and the UK) to contextually scrutinize the pool of items suggested by members of the first group and agree on an initial set of items. The principal investigator then developed a survey of the items approved by the second group. The survey developed was returned to the second expert group for review and approval. To pre-test the survey, the principal investigator engaged a third group of potential participants who were academics. Ten (10) hard copies of the survey were enveloped and sent to members of the third group through a private courier. The average age of this group was 37 years. Over 5 days, the survey was completed and returned through the courier by 9 out of the 10 academics. With a plain sheet provided by the researchers, the participants reported ambiguities and wording issues associated with the survey. Through a voice call, the principal investigator contacted participants who reported some issues to confirm and better understand the issues. This step enabled the lead researcher to improve the wording of the items. Major changes made to the survey were: (i) rearranging the items in a logical order; (ii) rewording questions relating to the demographic variables; and (iii) revising the preamble to the neighbourhood walkability scale to make it more concise. An online survey of the final items and the ethics statement was then constructed by one of the research team members and piloted online with 20 academics who were associates of the Africa Center for Epidemiology. Survey completion was done via WhatsApp (12 completed), email (4 completed), Facebook (2 completed) and LinkedIn (2 completed). With no issues reported in the second pilot study, we sent the survey back to the second group of experts to approve.

### Variables and their operationalization

The study involved six main variables, four of which were the job components (i.e. on-site teaching, online teaching, research and student assessment). The other two main variables were neighbourhood walkability and PA. Neighbourhood walkability was measured with the 11-item Australian version of the Neighbourhood Walkability Scale (NEWS-A). This measure has five descriptive anchors (i.e. strongly disagree—1; disagree—2; somewhat agree—3; agree—4; and strongly agree—5) and produced satisfactory psychometric properties in the general population (Merom *et al.*, 2015). We chose this scale over others because it is relatively short and was, therefore, more suited for online data collection. Other versions and scales were relatively long and had resulted in a high non-response in similar samples. The scale is transferable to Africa because it measures essential walkability factors shared by large and small cities and recently produced reliable results in Africa (Asiamah *et al.*, 2021). In the current study, it produced a Cronbach's α = 0.81. We generated an index on neighbourhood walkability by adding up its 11 items in harmony with standard practices (Floden and Bell, 2019). Supplementary Appendix A shows items of the neighbourhood walkability scale. PA was measured with the short form of the International Physical Activity Questionnaire (SF-IPAQ). The SF-IPAQ was used because it had produced satisfactory psychometric properties across the world's major populations and regions (Oyeyemi *et al.*, 2014; Wanner *et al.*, 2016; Lavelle *et al.*, 2020), including Africa (Oyeyemi *et al.*, 2014). Moreover, its scoring protocol was comprehensive and convenient. Table 1 shows how the job components and demographic variables were measured, coded and operationally defined. As the table indicates, categorical variables were dummy-coded. Demographic variables that had been evidenced in the literature to predict the job components, PA or neighbourhood walkability could confound the primary relationships and were, therefore, treated as potential covariates.

**Table 1:** Key measures, their operationalization and coding scheme

Category	Variable	Operational definition	Variable type	Coding	Dummy-coded?
Teaching	Teaching (online)	Time spent by the faculty member teaching online over the last 7 days	Continuous	—	No
	Teaching (on-site)	Time spent by the faculty member teaching in a physical classroom over the last 7 days	Continuous	—	No
Research	Research	The amount of time the individual had spent in the last 7 days on research-related activities	Continuous	—	No
Student assessment	Marking	The amount of time spent by the individual on marking of examination/test scripts in the last 7 days	Continuous	—	No
	Marks organization	The amount of time spent by the individual on organizing test/examination scores in the last 7 days	Continuous	—	No
	Quizzes and exams	The amount of time the individual spent on conducting quizzes and examinations in the last 7 days	Continuous	—	No
	Research supervision	The amount of time spent by the individual on student research project supervision in the last 7 days	Continuous	—	No
Demographic characteristics	Country	The country where the individual was working as a faculty member	Nominal	Nigeria (1); Ghana (2); Kenya (3); Tanzania (4)	Yes
	University category	Whether one's primary university was a public, private, or hybrid institution	Nominal	Public (1); private (2); hybrid^a^ (3)	Yes
	Gender	Sex of the individual	Nominal	Male (0); female (1)	Yes
	Education	The highest educational qualification of the individual	Ordinal	Diploma (1); first degree (2); Master's degree (3); PhD or equivalent (4)	No
	Income	The individual's gross monthly income in United States Dollars	Ordinal	0–500 (1); 501–1000 (2); 1001–1500 (3); 1501–2000 (4); above 2000 (5)	No
	Rank^b^	The academic rank of the individual	Ordinal	Teaching assistant (1); assistant lecturer (2); lecturer (3); senior lecturer (4); assistant professor (5); associate professor (6); full professor (7)	No
	Residency	Whether the individual was resident on the campus of his or her primary university	Nominal	No (0); Yes (1)	Yes
	Tenure	The number of years the individual had served as an academic	Continuous	—	No
	Alternative role(s)	Whether the individual had other academic job roles outside his/her primary institution	Nominal	No (0); Yes (1)	Yes
	CDS	Whether the individual had at least one clinically diagnosed chronic condition	Nominal	None (0); ≥1 (1)	Yes
	Age	The age of the individual in years	Continuous	—	No

a An institution with public and private ownership status.

b Each category was associated with its research specialization.

—, not applicable; CDS—chronic disease status.

### Study ethics and data collection

This study received ethical clearance from an institutional ethics review committee (# 0022020ACE) in Accra after the study protocol and ethical consent statement were reviewed by the committee. As part of the ethical steps taken, we emphasized with the first question the need for individuals to complete the survey voluntarily. The first question also stated the risk-free nature of our data collection process and the importance of the study. It included the selection criteria and assured participants that their participation was anonymized. The survey was published on 10 October 2020, by sending them to social media groups including or comprising academics. The link was shared on four WhatsApp and Telegram group pages managed by the Africa Center for Epidemiology. These groups comprised academics of all ranks and students undertaking research studies in various universities around Africa. Eleven (11) nationalities were featured in the group, but the majority of academics in the group came from Ghana, Nigeria, Kenya and Tanzania. The four groups contained a total of 361 members as of 10 October 2020. With support from these members, we employed the snowball selection process used by Asiamah *et al.* (Asiamah *et al.*, 2021) to distribute the questionnaire. Members who met the inclusion criteria on the platforms were implored to complete the survey and share it with colleagues in their universities and countries. Members were guided to distribute the survey ethically to maximize voluntary participation. All researchers further distributed the survey by posting it on their faculty social media platforms. The short link took the participants to a pop-up questionnaire that could be completed with a relatively weak internet network. Participants did not have to download the survey before completing it. The survey was distributed and completed over 5 weeks and closed on 15 November 2020. The survey's average completion time was about 5 min. We programmed the survey to prevent more than one response from the same participant. No incentives were provided to participants.

### Statistical analysis method

The data, in a default Microsoft Excel file, were downloaded from Google Forms and transported to SPSS version 25 (IBM Inc., NY, USA) where coding and data analysis were carried out. Descriptive statistics (i.e. frequencies and percentage points) were generated to assess the distribution of the data after 14 questionnaires with at least 10% of its variables associated with missing data were dropped in line with recommendations and previous practices (Garson, 2012; Asiamah *et al.*, 2021). The normality of the data associated with the main dependent variable (i.e. PA), which is a requirement for the use of parametric tests, was assumed and assessed with the Shapiro-Wilk's test. Results of this test met the condition *p* ≥ 0.05 recommended by Garson (Garson, 2012) and, therefore, evidenced the normality of the data. We employed the following standard formula from Lavelle *et al.*(Lavelle *et al.*, 2020) to generate the index on PA:

TotalMET-minutes/week=Walking(MET*min*days)+ModeratePA(MET*min*days)+VigorousPA(MET*min*days).

The acronym MET in the above equation stands for *Metabolic Equivalent* whereas walking, moderate PA and vigorous PA are the three main domains of the SF-IPAQ. The MET levels originally assigned to these three domains are as follows: walking = 3.3, moderate PA = 4 and vigorous PA = 8.

Before the primary relationships were evaluated, we carried out a sensitivity analysis recently used by Asiamah *et al.* (Asiamah *et al.*, 2020) to screen for relevant covariates. With this analysis, only important covariates or variables that could significantly affect the primary relationships were infused into the final analysis. In the first stage of this analysis, we developed an index of the four job components, after which univariate regression models were fitted to estimate crude coefficients (i.e. standardized and unstandardized coefficients and their 95% confidence intervals) that represent the effects of the job components on PA. At this stage, covariates with *p* > 0.25 were removed and those with *p* ≤ 0.25 were kept for the second stage. Country, university category, chronic disease status (CDS) and alternative role(s) were removed at this stage. At the second stage, a multiple linear regression model was fitted to estimate the effects (including 95% confidence intervals) of each job component and covariates (from Stage 1) on PA. Any covariate that led to a 10% change in the coefficients between the job components and PA was infused into two *ultimate models* as the ultimate covariate. The first ultimate model tested the primary relationships while adjusting for the ultimate covariates whereas the second one examined moderating influences and controlled for the ultimate covariates. Gender, education, income, rank and age were incorporated into the final analysis as the ultimate covariates.

In the main analysis, four hierarchical regression models were fitted to test the relevant relationships. The first model (i.e. the first baseline model) examined the regression coefficients between the job components and PA without controlling for the ultimate covariates. The second baseline model assessed the moderation influences without adjusting for the ultimate covariates. The third and fourth models represent the two ultimate models described above. As part of the sensitivity analysis, we compared the ultimate models with the baseline models to identify possible changes in their regression weights and significance values. This step enabled us to know if the primary relationships are affected by the ultimate covariates. As a basis for the moderation test, we followed the procedure of Helm and Mark (Helm and Mark, 2012) to compute four interaction terms: *teaching(*online)*NW (i.e. the interaction between teaching online and neighbourhood walkability), teaching(on-site)*NW (i.e. the interaction between teaching on-site and neighbourhood walkability), research*NW (i.e. the interaction between research and neighbourhood walkability), and assessment*NW (i.e. the interaction between student assessment and neighbourhood walkability). The relationship between each of these terms and PA was then assessed with the second baseline and ultimate models. The type of moderation tested was pure moderation (Helm and Mark, 2012), which means that we were interested only in how much neighbourhood walkability changed the regression weights between the job components and PA. Statistical significance of the result was detected at *p* < 0.05 and the ultimate models treated as the source of findings.

## RESULTS

A total of 1201 surveys were completed. After applying the selection criteria, 1,064 surveys were analysed. Tables 2 and 3 show summary statistics on participants. About 35% (*n *=* *372) of the participants were from Ghana, 38% (*n *=* *400) were from Nigeria, 26% (*n *=* *272) were from Kenya and 2% (*n *=* *20) were from Tanzania. About 68% (*n *=* *720) of the participants were men whereas 32% (*n *=* *344) were women. In Table 3, the average age of participants was about 44 years while the average PA was about 4,475 MET-minutes/week (Mean = 4475.3; SD = 2754.3). Supplementary Appendices B1 and B2 show results of an analysis of variance (ANOVA) performed to assess differences between the four countries in terms of the main variables (i.e. PA, the four job components and neighbourhood walkability). The ANOVA results in Supplementary Appendix B1 show that there was a difference between the countries in terms of each of the main variables. For instance, there was a significant difference in PA between the four countries (F = 3.76; *p *= 0.11). In Supplementary Appendix B2, the post-hoc analysis suggests that this difference was due to Ghana and Kenya having different PA levels (*p *= 0.007). Other paired differences can be found in Supplementary Appendix B2.

**Table 2:** Summary statistics on covariates and personal variables

Variable	Group	*n*	%
Country	Ghana	372	35.0
	Nigeria	400	37.6
	Kenya	272	25.6
	Tanzania	20	1.9
	Total	1064	100
University category	Public	760	71.4
	Private	280	26.3
	Hybrid (public and private)	24	2.3
	Total	1064	100
Gender	Male	720	67.7
	Female	344	32.3
	Total	1064	100
Education	Diploma	16	1.5
	First degree	76	7.1
	Master's degree	480	45.1
	PhD or equivalent	492	46.2
	Total	1064	100.0
Income (USD)	0–500	192	18.0
	501–1000	388	36.5
	1001–1500	224	21.1
	1501–2000	96	9.0
	Above 2000	164	15.4
	Total	1064	100
Rank^a^	Teaching assistant	96	9.0
	Assistant lecturer	192	18.0
	Lecturer	380	35.7
	Senior lecturer	248	23.3
	Associate professor	76	7.1
	Professor (full)	72	6.8
	Total	1064	100
Campus residency	No	880	82.7
	Yes	184	17.3
	Total	1064	100
Alternative role(s)	No	624	58.6
	Yes	440	41.4
	Total	1064	100
Chronic disease status	None	836	78.6
	≥1	228	21.4
	Total	1064	100

a Each category was associated with its research specialization.

n, frequency; USD, United States Dollars.

**Table 3:** The correlation between core job components, physical activity, neighbourhood walkability and the ultimate covariates

Variable	Mean	SD	1	2	3	4	5	6	7	8	9	10	11
1. Teaching (online) (h/week)	5.3	4.2	1	−0.231^**^	0.047	0.048	−0.095^**^	−0.048	−0.015	−0.137^**^	−0.006	−0.181^**^	−0.069^*^
2. Teaching (on-site) (h/week)	6.0	4.9		1	−0.033	−0.055	0.093^**^	0.064^*^	−0.102^**^	−0.095^**^	−0.035	−0.014	−0.05
3. Research (h/week)	2.3	1.1			1	0.972^**^	−0.208^**^	−0.748^**^	0.102^**^	0.067^*^	0.038	0.014	−0.069^*^
4. Assessment (h/week)	7.0	3.2				1	−0.215^**^	−0.779^**^	0.094^**^	0.068^*^	0.054	−0.024	−0.076^*^
5. NW	27.7	6.2					1	0.205^**^	0.011	−0.109^**^	−0.011	−0.04	0.053
6. PA (MET-min/week)	4475.3	2754.33						1	−0.127^**^	−0.140^**^	−0.067^*^	−0.03	0.014
7. Gender (reference—male)	—	—							1	0.097^**^	−0.03	−0.062^*^	0.012
8. Education	—	—								1	0.156^**^	0.589^**^	0.432^**^
9. Income (USD)	—	—									1	0.172^**^	0.192^**^
10. Rank	—	—										1	0.628^**^
11. Age	44.25	9.632											1

** *p* < 0.001;

* *p* < 0.05;

—, not applicable; USD, United States Dollars; NW, neighbourhood walkability; SD, standard deviation; MET, metabolic equivalent.

**Table 4:** The associ ation between core job components, physical activity and the ultimate covariates

Model	Predictor^c^	Coefficients			95% CI	Tolerance	Predictor^d^	Coefficients			95% CI	Tolerance
		B	SE	β(*t*)				B	SE	*β*(*t*)		
1^a^	(Constant)	9066.76	164.73	(55.04)^**^	±646.48		(Constant)	7563.01	193.5	(39.09)^**^	±759.38	
	Teaching (online) (h/week)	−4.87	12.89	−0.01 (−0.38)	±50.57	0.95	NW^*^Teaching (online)	146.4	55.44	0.06 (2.64)^*^	±217.58	0.98
	Teaching (on-site) (h/week)	9.24	11.07	0.02 (0.83)	±43.44	0.94	NW^*^Teaching (on-site)	275.92	34.15	0.19 (8.08)^**^	±134.00	0.97
	Research (h/week)	417.8	207.71	0.17 (2.01)^*^	±815.15	0.05	NW^*^Research	60.56	774.16	0.01 (0.08)	±3038.13	0.07
	Assessment (h/week)	−805.01	71.01	−0.94 (−11.34)^**^	±278.66	0.05	NW^*^Assessment	−1868.85	262.03	−0.65 (−7.13)^**^	±1028.32	0.07
	Model fit											
	* R* ^2^	0.609						0.431				
	* *Adjusted *R*^2^	0.607						0.429				
	* *Change in *R*^2^	0.002						0.002				
	* *Durbin–Watson	1.608						1.66				
	* *F-test	412.04^**^						200.72^**^				
2^b^	(Constant)	10 542.99	404.78	(26.05)^**^	±1588.61		(Constant)	8558.31	473.82	(18.06)^**^	±1859.55	
	Teaching (online) (h/week)	−10.29	13.28	−0.02 (−0.78)	±52.12	0.91	NW^*^Teaching (online)	130.67	57.33	0.06 (2.28)^*^	±225.00	0.95
	Teaching (on-site) (h/week)	−0.07	11.24	0.00 (−0.01)	±44.10	0.91	NW^*^Teaching (on-site)	249.92	34.47	0.17 (7.25)^**^	±135.27	0.96
	Research (h/week)	515.03	212.43	0.20 (2.42)^*^	±833.71	0.05	NW^*^Research	176.62	786.03	0.02 (0.23)	±3084.85	0.07
	Assessment (h/week)	−836.87	72.86	−0.97 (−11.49)^**^	±285.96	0.05	NW^*^Assessment	−1943.31	267.12	−0.65 (−7.28)^**^	±1048.33	0.07
	Covariates											
	* *Gender (reference—male)	−304.48	117.88	−0.05 (−2.58)^*^	±462.63	0.95	Gender (ref.—male)	−344.94	142.04	−0.06 (−2.43)^*^	±557.44	0.94
	* *Education	−251.09	108.28	−0.06 (−2.32)^*^	±424.95	0.61	Education	−366.87	130.21	−0.09 (−2.82)^*^	±511.00	0.61
	* *Income (USD)	−23.88	42.64	−0.01 (−0.56)	±167.33	0.95	Income (USD)	−12.07	51.39	−0.01 (−0.24)	±201.69	0.94
	* *Rank	−53.39	52.16	−0.03 (−1.31)	±204.70	0.46	Rank	−7.39	62.33	0.00 (−0.12)	±244.63	0.46
	* *Age	−5.55	7.44	−0.02 (−0.75)	±29.18	0.58	Age	11.32	8.91	0.04 (1.27)	±34.98	0.59
	Model fit											
	* R* ^2^	0.616						0.445				
	* *Adjusted *R*^2^	0.613						0.44				
	* *Change in *R*^2^	0.003						0.005				
	* *Durbin–Watson	1.602						1.66				
	* *F-test	180.78^**^						90.25^**^				

** *p* < 0.001;

* *p* < 0.05.

a Baseline models.

b Ultimate models.

c Primary relationships.

d Intervening relationships.

SE, standard error; CI, confidence interval; USD, United States Dollars; NW, neighbourhood walkability; dependent variable—physical activity.

A significant negative correlation between PA and research (*r* = −0.748; *p* = 0.000; two-tailed) as well as student assessment (*r* = −0.779; *p* = 0.000; two-tailed) was found. A positive correlation between PA and teaching (on-site) was also found (*r *=* *0.064; *p* < 0.05; two-tailed). The correlation between teaching online and PA was not significant (*r* = −0.048; *p* > 0.05; two-tailed). Last but not least. a positive relationship between neighbourhood walkability and PA was found (*r *=* *0.205; *p* = 0.000; two-tailed).

After controlling for the ultimate covariates (see Table 4), PA was found to be positively associated with research (β = 0.2; *t *=* *2.42; *p* < 0.05) but negatively associated with student assessment (β = −0.97; *t* = −11.49; *p* = 0.000). Thus, PA increases with research work but reduces with student assessment. PA was negatively associated with education, implying that PA decreases as education increases. Men reported larger PA scores compared with women. PA was positively associated with the interaction between neighbourhood walkability and online teaching (β = 0.06; *t *=* *2.28; *p* < 0.05) as well as neighbourhood walkability and on-site teaching (β = 0.17; *t *=* *7.25; *p* = 0.000). This result connotes that the relationship between PA and online teaching is made positively significant by neighbourhood walkability. Similarly, the positive relationship between research and PA is strengthened by neighbourhood walkability. The interaction between neighbourhood walkability and assessment was negatively associated with PA (β = −0.65; *t* = −7.28; *p* = 0.000). The first ultimate model produced a variance of 61.6% and a significant *F*-test (*F *=* *180.78; *p* = 0.000) while the second ultimate produced a variance of 44.5% and a significant *F*-test (*F *=* *90.25; *p* = 0.000). Both models produced Durbin–Watson statistics ranging between 1.5 and 2.4 as recommended in the literature (Garson, 2012), which satisfied the independence-of-errors assumption. Each predictor in the two ultimate models also met the recommended criterion tolerance ≥0.1 (Garson, 2012), which met the multi-collinearity assumption.

## DISCUSSION

This study assessed the relationship between key job components and PA among academics in a post-COVID-19 context in Africa. The moderating influence of neighbourhood walkability in the association between the job components and PA was also evaluated.

After controlling for the ultimate confounding variables, the study found a positive association between the job component research and PA, which suggests that an increase in research is associate with larger PA scores. Research among academics can take two main components, fieldwork and desk research, with the latter concerned with literature reviews and reading (Torp *et al.*, 2017, 2018) and, therefore, requires sedentary behaviours such as sitting. Fieldwork, on the other, requires social activities, walking and other physical activities in data collection and the supervision of research assistants and contractors. Thus, research may support PA, at least in part, if it encourages participation in field activities. Our evidence, therefore, calls for a reconsideration of the idea that on-site teaching is the only PA-oriented job tasks among academics. A positive association between research and PA is, thus, a potential outcome of research involving less sitting and more of social activities, walking and other physical activities in a post-COVID-19 context. Alternatively, academics in the post-COVID-19 period carried out more fieldwork involving walking and other physical activities in their studies, possibly because most of them had a backlog of field research tasks to perform at the time of reopening. The inability of academics to undertake field research due to previous social distancing measures (e.g. a lockdown) may have led to the said backlog. Owing to the ‘publish or perish’s culture that is growing remarkably in Africa (Yankholmes, 2014), academics would not hesitate to quickly clear or at least reduce their backlog of field research work after universities reopened. Further to this, academics may have spent a lot of time indoors doing desk research (i.e. literature reviews), the result of which would require primary data from the field. While the above thoughts are a possible way to explain our result, future research investigating why research may have a positive relationship with PA is needed to clarify our finding. With the above understanding, we infer that a negative association between research and PA would be the consequence of occupational sitting associated with reading, literature review and writing without active participation in fieldwork. Given our findings, it is likely that academics did little desk research and writing during the post-COVID-19 period. Since the IPAQ-SF does not report leisure-time PA, academics likely performed leisure-time PA.

Student assessment was found to be negatively associated with PA, which implies that PA decreases as student assessment increases. This result is consistent with commentators (Hogan *et al.*, 2016; Booket *et al.*, 2018; Egan *et al.*, 2019) who have described student assessment as a sedentary job component. Egan *et al.* (Egan *et al.*, 2019), for example, insinuated that marking and organization of test results require significant occupational sitting. Booket *et al.* (2018) also acknowledged that exam supervision is PA-oriented because it involves classroom activities (e.g. invigilation) or social engagement with students, but the amount of time spent in script marking and scores organization is significantly more than time spent in invigilation and similar activities. Corroborating this argument is Hogan *et al.*(Hogan *et al.*, 2016) who observed that the supervision of internships and student fieldwork is the only significant PA-oriented component of student assessment among academics; however, academics in Africa were not involved in this form of assessment in a post-COVID-19 period when academics and students were still observing social distancing protocols (Adarkwah, 2020; Harrington and O’Reilly, 2020). As such, there was no need to measure fieldworks associated with student assessment at a time academics were observing social distancing and did not have students in the field to supervise. In light of the above thoughts and results, it is understandable that academics in the post-COVID-19 context spent more sedentary time on student assessment in the way of marking and organizing marks from tests and exams. It is also possible that physical invigilation was replaced with online student monitoring and engagement.

A more compelling result could be the moderating influence of neighbourhood walkability on the relationship between the job components and PA. That is, neighbourhood walkability made the non-significant negative relationship between online teaching and PA positively significant. It also weakened the negative association between student assessment and PA and increased the strength of the positive association between research and PA. These results imply that neighbourhood walkability buffers sedentariness associated with the core job components. Backing this role of neighbourhood walkability in the relationships is Lawton's (Lawton, 1980) Person-Environment (P-E) fit model, which posits that human behaviours (e.g. PA) in a community are influenced by the built environment including essential services and amenities. The model adds that services, parks, community centres, aesthetics and road networks are neighbourhood resources that would often encourage social activities, walking and other physical activities. The study of Hosler *et al.*(Hosler *et al.*, 2014) supports the above theory with its positive relationship between neighbourhood walkability and active behaviours in older adults with diabetes. In their scoping review, Edwards and Dulai (Edwards and Dulai, 2018) revealed consistent confirmation of the positive relationship between neighbourhood walkability and physical activity. In Nigeria, Oyeyemi *et al.*(Oyeyemi *et al.*, (2019) found a positive association between PA and neighbourhood walkability. Asiamah *et al.* (Asiamah *et al.*, 2020) confirmed a positive association between walkable resources in the community and social activities. Since academics had the opportunity to access walkable resources in their communities after the lockdown or the reopening of universities, our results and these pieces of evidence suggest that neighbourhoods played a role in buffering sedentary behaviour and increasing PA.

In our sample, men reported higher PA scores compare with women, which suggests that men were possibly more active in a post-COVID-19 context than women. Though this result is consistent with some studies conducted in different contexts (Mutlu *et al.*, 2015; Asiamah, 2016), there are studies that reported higher PA scores for women (Lee *et al.*, 2011; Cleland *et al.*, 2018). We observed that these mixed pieces of evidence based on the IPAQ-SF were due to variations in context and research design. Furthermore, our average PA score of 4475 MET min/week is higher than most averages reported in similar studies conducted in developed countries (Violant-Holz *et al.*, 2020) and in studies conducted in a COVID-19 social distancing context (Violant-Holz *et al.*, 2020). We have two explanations for this result. First, Asiamah *et al.* (Asiamah *et al.*, 2021) reasoned that PA might have increased in COVID-19 social distancing contexts because exercise and PA were promoted as a way to build immunity against COVID-19. Supporting this reasoning is a pilot study conducted in a COVID-19 social distancing context in Tunisia that reported an average IPAQ-SF-based PA of approximately 6074 MET min/week (Slimani *et al.*, 2020). This average came from individuals of the general population who exercised to enhance health during the pandemic. Secondly, Guthold *et al.*(Guthold *et al.*, 2018) reported that African countries have the least levels of PI insufficiency, which means that a high level of PA in Africa during social distancing can be expected.

The second sensitivity analysis in which ultimate confounding variables were controlled for by comparing the baseline and ultimate models unfolds several implications and lessons. Firstly, personal demographic variables can affect the relationship between the job components and PA as well as the moderating role of neighbourhood walkability in the relationship between the job components and PA. Supporting this reasoning is the P-E fit model of Lawton (Lawton, 1980) that asserts that human behaviour is influenced by the environment and personal factors such as income, gender, and educational level. Some occupational health experts (Booket *et al.*, 2018; Hatipoglu and Inelmen, 2018) have also reported the link between employee behaviour and personal factors. The P-E fit model also asserts that PA and other behaviours are a function of individual factors and the environment, a reason why it was necessary to adjust for the ultimate confounding variables. A key implication is that it is always necessary for researchers to identify and control for confounding variables that could cause an over- or under-estimation of effects. As Asiamah *et al.* (Asiamah *et al.*, 2019) demonstrated in their study, however, confounding is not always possible, a reason why researchers must conduct robust literature reviews to identify potential confounders for their studies and screen for ultimate covariates using a robust statistical procedure.

Our results have several implications for practice, theory and research. Firstly, this study re-emphasizes the role of the built environment, particularly neighbourhood walkability, in the promotion of PA among academics and the general population. This is to say that the provision of walkable neighbourhoods can give academics and other residents access to services and resources that encourage walking, bicycling, and other forms of active transportation. Walkable neighbourhoods can, therefore, encourage PA in a post-pandemic context where social distancing measures are taken while community resources (including essential services) are being utilized. If parks and recreational centres (which form the core of neighbourhood walkability) could support individual-level PA during the lockdown (Asiamah *et al.*, 2021), then they should be expected to do more in a post-pandemic context with improved neighbourhood sociability. Residents may, nevertheless, need to be trained to use walkable neighbourhoods in a post-pandemic context since walkable neighbourhoods and social networks within them can have limited usability in this context. That being so, training and human development programs that enable academics, especially the older ones, to use walkable neighbourhoods are necessary. This is another way to say that even the most walkable neighbourhoods would not support PA or buffer sedentary behaviour in the general population if residents are not trained to safely navigate neighbourhood barriers (i.e. poor roads and hilly paths) in a post-COVID-19 setting. More so, residents may not use walkable resources if they are not made aware of the health and social benefits of such resources. The provision of walkable neighbourhoods, which is a public health flagship program in the developed world (Hosler *et al.*, 2014; Oyeyemi *et al.*, 2019), and the empowerment of individuals to utilize such neighbourhoods should, therefore, be the way forward in public health promotion in African countries.

This study has some limitations that have major implications for future research. The first limitation is our use of the cross-sectional approach, which did not allow us to establish cause and effect. This limitation hinges on the inability of cross-sectional designs to eliminate confounding completely (Asiamah *et al.*, 2019). The IPAQ-SF questionnaire used does not distinguish between PA during work and leisure; therefore, this study does not draw conclusions about the extent of PA during work and leisure. More so, we could not measure PA due to desk research and field work to back our argument. Future researchers are encouraged to provide methods and tools for measuring work-related PA, including PA related to desk research and fieldwork.

For reasons already discussed, our measure of student assessment did not include fieldwork evaluation (e.g. internship assessment). Future researchers are, as a result, expected to incorporate this indicator into student assessment if their contexts support it. The use of a non-powered sample and snowball sampling due to research design constraints could be associated with sampling bias and may, therefore, limit the generalizability of our findings. This notwithstanding, our geographic scope and sample size are sufficiently large and could provide findings reflecting an African context, at least. More importantly, our sensitivity analysis, apart from enabling us to remove confounding and irrelevant covariates in the ultimate model, can be a model for future researchers, considering the fact that this statistical technique is not commonly used. The inferior but dominant tradition has been the failure of researchers to control for confounders and remove irrelevant covariates in their regression models using standard statistical procedures such as our sensitivity analysis (Rothman and Greenland, 1998; Asiamah *et al.*, 2019). Our study, in effect, can encourage the application of a more resilient method for controlling for potential confounders in future research. Additional analyses comparing the estimated associations between the four countries would have been useful in this study, but these analyses were not performed for two main reasons. Firstly, this comparative analysis would have made the study more complex, making its scope difficult to understand and appreciate. Secondly, the primary goal of this study was to provide evidence on an African sample and set basis for future research. Thus, based on our results, future researchers can compare our associations between African countries.

## CONCLUSION

Research work as a job component was associated with higher PA among academics but student assessment was associated with lower PA. It is, therefore, concluded that PA among African academics could reduce as student assessment in the post-COVID-19 context increases. More compelling is the positive moderating influences of neighbourhood walkability on the relationship between the core job components and PA. More specifically, the non-significant relationship between online teaching and PA is made positively significant by neighbourhood walkability. The positive relationship between on-site teaching and PA is strengthened by neighbourhood walkability whereas the negative association between student assessment and PA is made less negative by neighbourhood walkability. It is concluded that access to walkable neighbourhoods can encourage PA and reduce sedentary behaviour as academics accomplish core job tasks in a post-COVID-19 context. Neighbourhood walkability can predict higher PA even among academics performing sedentary job tasks.

Our findings corroborate the need for governments, ministries of education, and universities in Africa to roll out interventions that support PA among academics. These interventions may include investments in more walkable university campuses and neighbourhoods, workplace PA programs (e.g. the provision of more equipped gyms and sporting facilities), and institutionalized campaigns aimed at encouraging academics to maintain PA and meet recommended PA levels. There is a need for future researchers to identify context-specific interventions by (i) using qualitative approaches to understand factors that may affect PA among academics in specific settings and (ii) applying prospective and cross-sectional designs to assess the cost-effectiveness of potential PA interventions and long-term effects of PA.

## Supplementary material

Supplementary material is available at *Health Promotion International* online.

## ETHICAL APPROVAL

This study received ethical clearance from an institutional ethics committee (# 0022020ACE). The ethics committee reviewed the study protocol and the informed consent statement. All participants consented to participate in this study.

## AUTHORS’ CONTRIBUTIONS

N.A. conceived the research idea, analysed the data and wrote the original manuscript. FFO designed the online survey and coordinated data gathering. F.M. coordinated data gathering in Kenya whereas E.D. contributed to the refinement of the research idea. S.M.A. and I.A. facilitated the distribution of the online survey. A.O. coordinated the distribution of the online survey in Nigeria. H.K.M. contributed to the study design and conceptualization. S.H., R.S.B., E.E. and C.A.M. coordinated questionnaire distribution. All authors proofread the draft manuscript and suggested corrections.

## Supplementary Material

daab090_Supplementary_Data
