# Supplementary material for: The relationship between job components, neighbourhood walkability and African academics' physical activity: a post-COVID-19 context
Source: Health Promot Int. 2021 Jul 19;37(2):daab090. doi: 10.1093/heapro/daab090 (PMC8411380; doi:10.1093/heapro/daab090)
Supplement: daab090_Supplementary_Data [file daab090_supplementary_data.zip › daab090-suppl_data/Appendix A.doc]

Appendix A. Items of Neighbourhood Sociability

|  | Item | 1 | 2 | 3 | 4 | 5 |
| --- | --- | --- | --- | --- | --- | --- |
| 1 | Many places are easy to go within walking distance |  |  |  |  |  |
| 2 | It is easy to walk to a public transport stop |  |  |  |  |  |
| 3 | There are footpaths along most of the streets |  |  |  |  |  |
| 4 | There are crosswalks and pedestrian signals |  |  |  |  |  |
| 5 | The streets in my neighbourhood are not hilly |  |  |  |  |  |
| 6 | Walkers in my neighbourhood can easily be seen |  |  |  |  |  |
| 7 | There is lots of greenery around my neighbourhood |  |  |  |  |  |
| 8 | There are many interesting things to look at |  |  |  |  |  |
| 9 | There is not much traffic along nearby streets |  |  |  |  |  |
| 10 | My neighbourhood has parks and walking trails |  |  |  |  |  |
| 11 | Crime rate in my neighbourhood is not a problem |  |  |  |  |  |

¶This table shows items of the Australian version of the Neighbourhood Environment Walkability Scale (NEWS-A). This scale was wholly borrowed from Merom et al. (2015); Descriptive anchors: 1 = strongly disagree; 2 = disagree; 3 = somewhat agree; 4 = agree, and 5 = strongly agree.
