# Supplementary material for: The relationship between job components, neighbourhood walkability and African academics' physical activity: a post-COVID-19 context
Source: Health Promot Int. 2021 Jul 19;37(2):daab090. doi: 10.1093/heapro/daab090 (PMC8411380; doi:10.1093/heapro/daab090)
Supplement: daab090_Supplementary_Data [file daab090_supplementary_data.zip › daab090-suppl_data/Appendix B1.doc]

Appendix B1. ANOVA test for differences between countries

| Variable | Country | N | Mean | SD | ANOVA | | EVA |
| --- | --- | --- | --- | --- | --- | --- | --- |
|  |  |  |  |  | F | Sig. |  |
| Teaching (online) | Ghana | 372 | 5.25 | 4.497 | 31.01 | 0.000 | No |
|  | Nigeria | 400 | 4.12 | 2.293 |  |  |  |
|  | Kenya | 272 | 7.19 | 5.333 |  |  |  |
|  | Tanzania | 20 | 5.6 | 2.798 |  |  |  |
|  | Total | 1064 | 5.33 | 4.226 |  |  |  |
| Teaching (on-site) | Ghana | 372 | 5.78 | 4.278 | 20.83 | 0.000 | No |
|  | Nigeria | 400 | 6.66 | 5.3 |  |  |  |
|  | Kenya | 272 | 4.66 | 3.739 |  |  |  |
|  | Tanzania | 20 | 12.2 | 11.954 |  |  |  |
|  | Total | 1064 | 5.95 | 4.941 |  |  |  |
| Research | Ghana | 372 | 2.25 | 1.066 | 2.72 | 0.043 | No |
|  | Nigeria | 400 | 2.3 | 1.155 |  |  |  |
|  | Kenya | 272 | 2.49 | 1.066 |  |  |  |
|  | Tanzania | 20 | 2.2 | 0.768 |  |  |  |
|  | Total | 1064 | 2.33 | 1.098 |  |  |  |
| Assessment | Ghana | 372 | 6.74 | 3.197 | 4.46 | 0.004 | No |
|  | Nigeria | 400 | 6.74 | 3.298 |  |  |  |
|  | Kenya | 272 | 7.56 | 3.109 |  |  |  |
|  | Tanzania | 20 | 6.6 | 2.303 |  |  |  |
|  | Total | 1064 | 6.95 | 3.215 |  |  |  |
| Neighbourhood walkability | Ghana | 372 | 28.8 | 5.91 | 6.61 | 0.000 | No |
|  | Nigeria | 400 | 26.85 | 5.922 |  |  |  |
|  | Kenya | 272 | 27.53 | 6.368 |  |  |  |
|  | Tanzania | 20 | 27 | 11.107 |  |  |  |
|  | Total | 1064 | 27.71 | 6.213 |  |  |  |
| Physical activity | Ghana | 372 | 4766.71 | 2991.88 | 3.76 | 0.011 | No |
|  | Nigeria | 400 | 4463.37 | 2674.6 |  |  |  |
|  | Kenya | 272 | 4055.84 | 2528.1 |  |  |  |
|  | Tanzania | 20 | 4998.60 | 1947.19 |  |  |  |
|  | Total | 1064 | 4475.31 | 2754.33 |  |  |  |

**Note**: SD – standard deviation; ANOVA – Analysis of Variance; EVA – Equal variances assumed; No indicates that EVA is not met at p >0.05.
