# Supplementary material for: The relationship between job components, neighbourhood walkability and African academics' physical activity: a post-COVID-19 context
Source: Health Promot Int. 2021 Jul 19;37(2):daab090. doi: 10.1093/heapro/daab090 (PMC8411380; doi:10.1093/heapro/daab090)
Supplement: daab090_Supplementary_Data [file daab090_supplementary_data.zip › daab090-suppl_data/Appendix B2.doc]

Appendix B2. Multiple Comparison (Post-hoc) Test

| Dependent Variable | (I) Country | (J) Country | MD (I-J) | Std. Error | Sig. | 95% CI | |
| --- | --- | --- | --- | --- | --- | --- | --- |
|  |  |  |  |  |  | LB | UB |
| Teaching (online) | Ghana | Nigeria | 1.127* | 0.29 | 0.001 | 0.35 | 1.90 |
|  |  | Kenya | -1.944* | 0.32 | 0.000 | -2.80 | -1.09 |
|  |  | Tanzania | -0.353 | 0.93 | 1.000 | -2.81 | 2.11 |
|  | Nigeria | Ghana | -1.127* | 0.29 | 0.001 | -1.90 | -0.35 |
|  |  | Kenya | -3.071* | 0.32 | 0.000 | -3.91 | -2.23 |
|  |  | Tanzania | -1.48 | 0.93 | 0.670 | -3.94 | 0.98 |
|  | Kenya | Ghana | 1.944* | 0.32 | 0.000 | 1.09 | 2.80 |
|  |  | Nigeria | 3.071* | 0.32 | 0.000 | 2.23 | 3.91 |
|  |  | Tanzania | 1.591 | 0.94 | 0.545 | -0.89 | 4.08 |
|  | Tanzania | Ghana | 0.353 | 0.93 | 1.000 | -2.11 | 2.81 |
|  |  | Nigeria | 1.48 | 0.93 | 0.670 | -0.98 | 3.94 |
|  |  | Kenya | -1.591 | 0.94 | 0.545 | -4.08 | 0.89 |
| Teaching (on-site) | Ghana | Nigeria | -0.875 | 0.35 | 0.070 | -1.79 | 0.04 |
|  |  | Kenya | 1.123* | 0.38 | 0.021 | 0.11 | 2.14 |
|  |  | Tanzania | -6.415* | 1.10 | 0.000 | -9.33 | -3.50 |
|  | Nigeria | Ghana | 0.875 | 0.35 | 0.070 | -0.04 | 1.79 |
|  |  | Kenya | 1.998* | 0.38 | 0.000 | 1.00 | 3.00 |
|  |  | Tanzania | -5.540* | 1.10 | 0.000 | -8.45 | -2.63 |
|  | Kenya | Ghana | -1.123* | 0.38 | 0.021 | -2.14 | -0.11 |
|  |  | Nigeria | -1.998* | 0.38 | 0.000 | -3.00 | -1.00 |
|  |  | Tanzania | -7.538* | 1.11 | 0.000 | -10.48 | -4.59 |
|  | Tanzania | Ghana | 6.415* | 1.10 | 0.000 | 3.50 | 9.33 |
|  |  | Nigeria | 5.540* | 1.10 | 0.000 | 2.63 | 8.45 |
|  |  | Kenya | 7.538* | 1.11 | 0.000 | 4.59 | 10.48 |
| Research | Ghana | Nigeria | -0.053 | 0.08 | 1.000 | -0.26 | 0.16 |
|  |  | Kenya | -.238* | 0.09 | 0.039 | -0.47 | -0.01 |
|  |  | Tanzania | 0.047 | 0.25 | 1.000 | -0.62 | 0.71 |
|  | Nigeria | Ghana | 0.053 | 0.08 | 1.000 | -0.16 | 0.26 |
|  |  | Kenya | -0.185 | 0.09 | 0.190 | -0.41 | 0.04 |
|  |  | Tanzania | 0.1 | 0.25 | 1.000 | -0.56 | 0.76 |
|  | Kenya | Ghana | .238* | 0.09 | 0.039 | 0.01 | 0.47 |
|  |  | Nigeria | 0.185 | 0.09 | 0.190 | -0.04 | 0.41 |
|  |  | Tanzania | 0.285 | 0.25 | 1.000 | -0.39 | 0.96 |
|  | Tanzania | Ghana | -0.047 | 0.25 | 1.000 | -0.71 | 0.62 |
|  |  | Nigeria | -0.1 | 0.25 | 1.000 | -0.76 | 0.56 |
|  |  | Kenya | -0.285 | 0.25 | 1.000 | -0.96 | 0.39 |
| Assessment | Ghana | Nigeria | 0.002 | 0.23 | 1.000 | -0.61 | 0.61 |
|  |  | Kenya | -.817* | 0.26 | 0.008 | -1.49 | -0.14 |
|  |  | Tanzania | 0.142 | 0.73 | 1.000 | -1.80 | 2.08 |
|  | Nigeria | Ghana | -0.002 | 0.23 | 1.000 | -0.61 | 0.61 |
|  |  | Kenya | -.819* | 0.25 | 0.007 | -1.48 | -0.15 |
|  |  | Tanzania | 0.14 | 0.73 | 1.000 | -1.80 | 2.08 |
|  | Kenya | Ghana | .817* | 0.26 | 0.008 | 0.14 | 1.49 |
|  |  | Nigeria | .819* | 0.25 | 0.007 | 0.15 | 1.48 |
|  |  | Tanzania | 0.959 | 0.74 | 1.000 | -1.00 | 2.92 |
|  | Tanzania | Ghana | -0.142 | 0.73 | 1.000 | -2.08 | 1.80 |
|  |  | Nigeria | -0.14 | 0.73 | 1.000 | -2.08 | 1.80 |
|  |  | Kenya | -0.959 | 0.74 | 1.000 | -2.92 | 1.00 |
| Neighbourhood walkability | Ghana | Nigeria | 1.946* | 0.44 | 0.000 | 0.77 | 3.12 |
|  |  | Kenya | 1.266 | 0.49 | 0.061 | -0.03 | 2.57 |
|  |  | Tanzania | 1.796 | 1.42 | 1.000 | -1.94 | 5.54 |
|  | Nigeria | Ghana | -1.946* | 0.44 | 0.000 | -3.12 | -0.77 |
|  |  | Kenya | -0.679 | 0.48 | 0.967 | -1.96 | 0.60 |
|  |  | Tanzania | -0.15 | 1.41 | 1.000 | -3.88 | 3.58 |
|  | Kenya | Ghana | -1.266 | 0.49 | 0.061 | -2.57 | 0.03 |
|  |  | Nigeria | 0.679 | 0.48 | 0.967 | -0.60 | 1.96 |
|  |  | Tanzania | 0.529 | 1.43 | 1.000 | -3.25 | 4.30 |
|  | Tanzania | Ghana | -1.796 | 1.42 | 1.000 | -5.54 | 1.94 |
|  |  | Nigeria | 0.15 | 1.41 | 1.000 | -3.58 | 3.88 |
|  |  | Kenya | -0.529 | 1.43 | 1.000 | -4.30 | 3.25 |
| Physical activity | Ghana | Nigeria | 303.3419 | 197.62 | 0.751 | -219.02 | 825.70 |
|  |  | Kenya | 710.87761* | 218.88 | 0.007 | 132.32 | 1289.44 |
|  |  | Tanzania | -231.887 | 629.77 | 1.000 | -1896.52 | 1432.75 |
|  | Nigeria | Ghana | -303.342 | 197.62 | 0.751 | -825.70 | 219.02 |
|  |  | Kenya | 407.5357 | 215.62 | 0.354 | -162.41 | 977.48 |
|  |  | Tanzania | -535.229 | 628.65 | 1.000 | -2196.89 | 1126.43 |
|  | Kenya | Ghana | -710.87761* | 218.88 | 0.007 | -1289.44 | -132.32 |
|  |  | Nigeria | -407.536 | 215.62 | 0.354 | -977.48 | 162.41 |
|  |  | Tanzania | -942.765 | 635.65 | 0.830 | -2622.94 | 737.41 |
|  | Tanzania | Ghana | 231.8871 | 629.77 | 1.000 | -1432.75 | 1896.52 |
|  |  | Nigeria | 535.229 | 628.65 | 1.000 | -1126.43 | 2196.89 |
|  |  | Kenya | 942.7647 | 635.65 | 0.830 | -737.41 | 2622.94 |

*Difference significant at p< 0.05; MD – mean difference; LB – lower bound; UP – upper bound; CI = confidence interval
